# Evaluation of collection efficiency and safety of a precise distention fluid collection device compared to traditional devices in hysteroscopic surgery

**DOI:** 10.3389/fsurg.2026.1894370

**Published:** 2026-10-07

**Authors:** Yongli Ying, Haiyan Lan

**Affiliations:** 1 Department of Operation Room, The First Affiliated Hospital of Lishui University, Lishui People's Hospital, Lishui, Zhejiang, China; 2 Department of Anesthesiology, The First Affiliated Hospital of Lishui University, Lishui People's Hospital, Lishui, Zhejiang, China

**Keywords:** distension fluid collection, fluid management, fluid overload, hysteroscopic surgery, operative hysteroscopy intravascular absorption syndrome

## Abstract

**Objective:**

This study aims to validate the effectiveness of this device in collecting distension fluid during hysteroscopic surgery, providing a more reliable tool for monitoring fluid outflow.

**Methods:**

A total of 164 patients scheduled for elective hysteroscopy were randomly assigned in a 1:1 ratio to either the precision collection group or the control group. The precision collection group utilized a precision distension fluid collection device for hysteroscopic distension fluid collection, while the control group used a conventional collection device. The primary outcome was the hysteroscopic distension fluid collection rate. Secondary outcomes included the incidence of adverse events, operating room cleaning time, and satisfaction levels of surgeons, nurses, and operating room support staff.

**Results:**

There were no significant differences in baseline patient characteristics or surgical types between the two groups. Compared to the conventional fluid collection device, the precision distension fluid collection device significantly improved the hysteroscopic distension fluid collection rate (95% vs. 48%, *P* < 0.001) and reduced the operating room turnover time for staff (4.1 min vs. 6.4 min, *P* < 0.001), while also substantially increasing surgical team satisfaction.

**Conclusion:**

The precision distension fluid collection device effectively enhances the collection rate of hysteroscopic distension fluid, reduces risks associated with improper fluid management, shortens operating room turnover time, and improves the satisfaction of the surgical team.

**Clinical Trial Registration:**

https://www.chictr.org.cn, identifier ChiCTR2500101460.

## Introduction

Hysteroscopy is the preferred method for diagnosing and treating intrauterine diseases in minimally invasive gynecological surgery and is recognized as the “gold standard” for diagnosing intrauterine lesions (1). Minimally invasive surgery conducted under the guidance of a visual screen through hysteroscopy has the advantages of less trauma, protection of normal tissues, no need for laparotomy, no interference with the function of pelvic organs, short operation time and fast postoperative recovery, and has become a routine technology in the field of gynecology. Since its first report in 1869, its diagnostic and therapeutic role has grown increasingly prominent; however, potential complications have also emerged (2). Although hysteroscopy is generally safe and some operations can even be completed in outpatient settings, high vigilance is required for severe complications, including fluid overload, uterine perforation, massive hemorrhage, infection, and air embolism (2–9) Among these, operative hysteroscopy intravascular absorption syndrome (OHIAS) is a severe complication in hysteroscopic surgery induced by the excessive absorption of distension media, with an incidence rate of 0.1% to 0.2% (10). This syndrome is primarily driven by two core pathophysiological mechanisms: fluid overload and dilutional hyponatremia. While mild cases carry a favorable prognosis with timely intervention, severe cases can rapidly progress to acute heart failure, fulminant pulmonary edema, and severe cerebral edema driven by acute fluid overload and profound hyponatremia, potentially leading to fatal outcomes (11). Consequently, rigorous intraoperative prevention and proactive management are of paramount clinical importance (12).

Hysteroscopic surgery necessitates distension media to expand the uterine cavity and maintain a clear surgical field. However, intraoperative distension pressure and media absorption carry inherent risks of fluid overload, hemodilution, and reduced plasma osmolality. Excessive fluid absorption remains an unavoidable challenge in hysteroscopic surgery, influenced by surgical modality, operative duration, distension pressure, media selection, and the exposure of deep myometrial venous sinuses resulting from tissue damage (13, 14). Current preventive strategies focus on utilizing bipolar electrosurgical systems (15), selecting appropriate distension media (10, 16), optimizing dilatation pressure (17, 18), and refining surgical techniques to minimize operative time.

Despite these preventive measures, precise monitoring of fluid inflow and outflow, and thereby estimating the net volume absorbed, remains the ultimate safeguard for identifying clinically actionable thresholds and preventing fluid overload. This is particularly critical when tissue damage extends into the deeper myometrium or breaches myometrial venous sinuses and large vessels, where an intrauterine pressure exceeding the venous or mean arterial pressure drives rapid, large-volume intravasation of distension fluid into the systemic circulation (10). Studies have shown that absorbing as little as 500 mL of non-electrolyte fluid can cause severe consequences, including fatal cerebral edema (19). However, current clinical methodologies for calculating fluid deficit lack precision (20). Circulating nurses must estimate the volume used from distension bags, the amount recovered in collection devices, and the fluid spilled onto sterile drapes or the floor, leading to significant measurement errors (21, 22).

To address this issue, our research team has developed a precision distension fluid collection device designed to efficiently capture fluid outflow from the hysteroscope, sheath gaps, and connection ports, thereby enabling precise calculation of distension fluid inflow and outflow. This study aims to validate the effectiveness of this device in collecting distension fluid during hysteroscopic surgery, providing a more reliable tool for monitoring fluid outflow. By facilitating accurate collection and monitoring of distension fluid, this device is expected to enhance the early recognition of, intervention for, and management of fluid overload, thereby improving the safety of hysteroscopic procedures, and offering a potentially valuable technical tool to assist in fluid monitoring during minimally invasive gynecological surgery.

## Methods and technologies for fluid output monitoring

### Precision distension fluid collection device method

The precision hysteroscopic fluid collection device is designed as a funnel-shaped collection bag (see Figures 1 and 2). The device consists of two surfaces, A and B, as shown in Figure 1. Surface A is a measuring bag equipped with scale markings to accurately quantify the liquid inside. The bottom of the collection bag is fitted with a sealing clip to facilitate the drainage of distension fluid and the calculation of fluid volume, with the bag placed above a graduated measuring bucket. The collection bag's opening is equipped with a support ring that can be shaped according to the surgeon's requirements, enabling continuous fluid collection from the hysteroscopic sheath interface. A filter mesh is attached below the support ring, adapting to changes in the bag's opening shape, to capture small tissue specimens discharged from the uterine cavity during surgery, preventing specimen loss.

**Figure 1:**
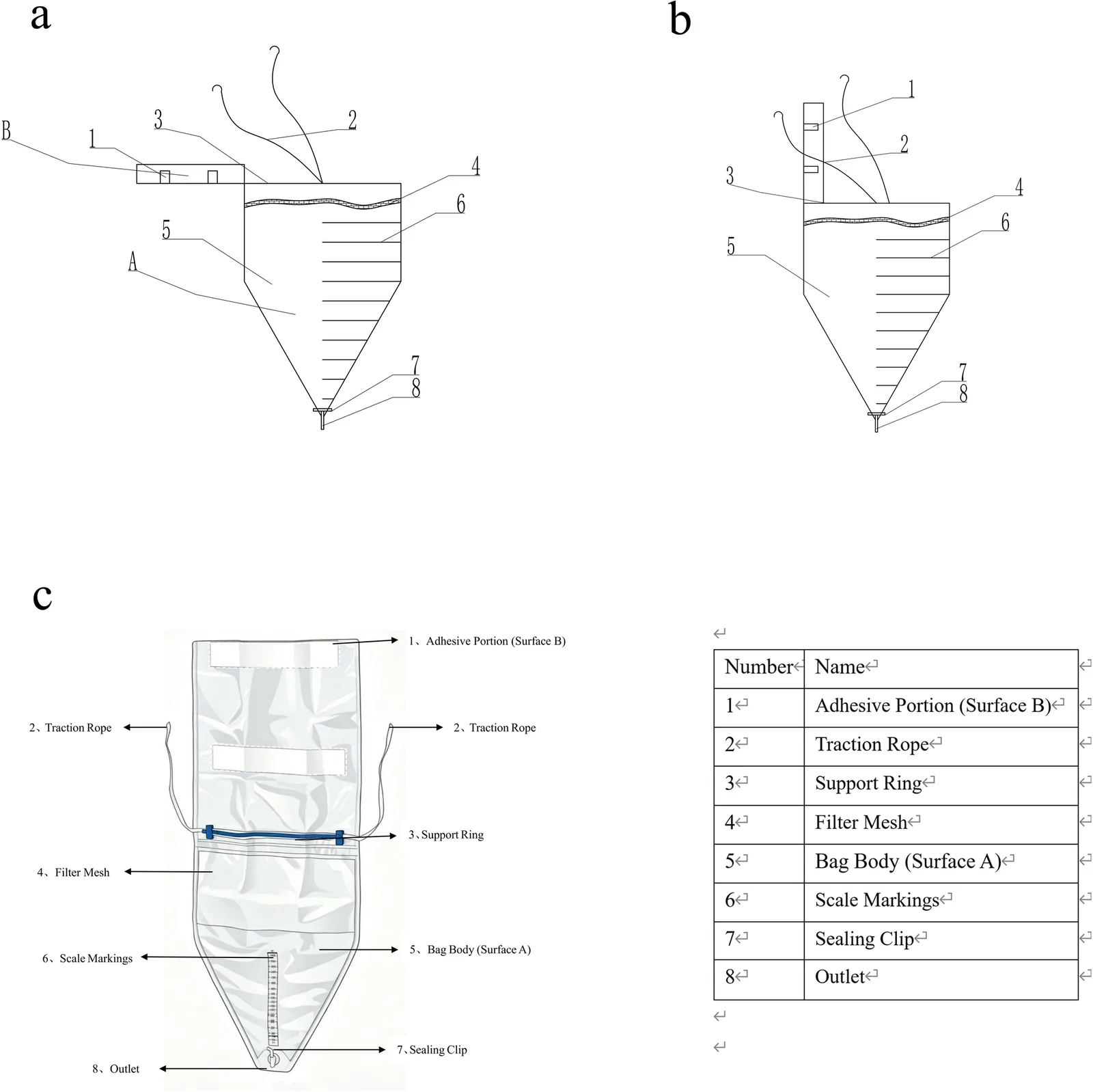
Precision distension fluid collection device. (a, b) Side–view schematic diagrams of the collection device. (c) Front–view schematic diagram of the collection device. Part labels (1–8): Part labels (1–8): 1, Adhesive Portion (Surface B), the adhesive area on Surface B that adheres to the bed to secure the collection bag; 2, Traction Rope, symmetrically arranged fixation ropes on both sides of the collection bag, tied to both sides of the stirrup frame in the lithotomy position to prevent tilting or detachment during fluid collection and ensure structural stability; 3, Support Ring, located at the opening of the collection bag and shapeable as needed during surgery to support the bag opening; 4, Filter Mesh, positioned below the support ring and adaptable to the shape of the bag opening, used to capture small tissue specimens discharged during surgery; 5, Bag Body (Surface A), the measuring bag with scale markings used for collecting and measuring distension fluid; 6, Scale Markings, provided on the measuring bag (Surface A) for accurate quantification of the collected fluid volume; 7, Sealing Clip, positioned at the bottom of the collection bag, used to control drainage of distension fluid and facilitate volume calculation; 8, Outlet, the fluid discharge outlet at the bottom of the collection bag, used in conjunction with the sealing clip.

**Figure 2:**
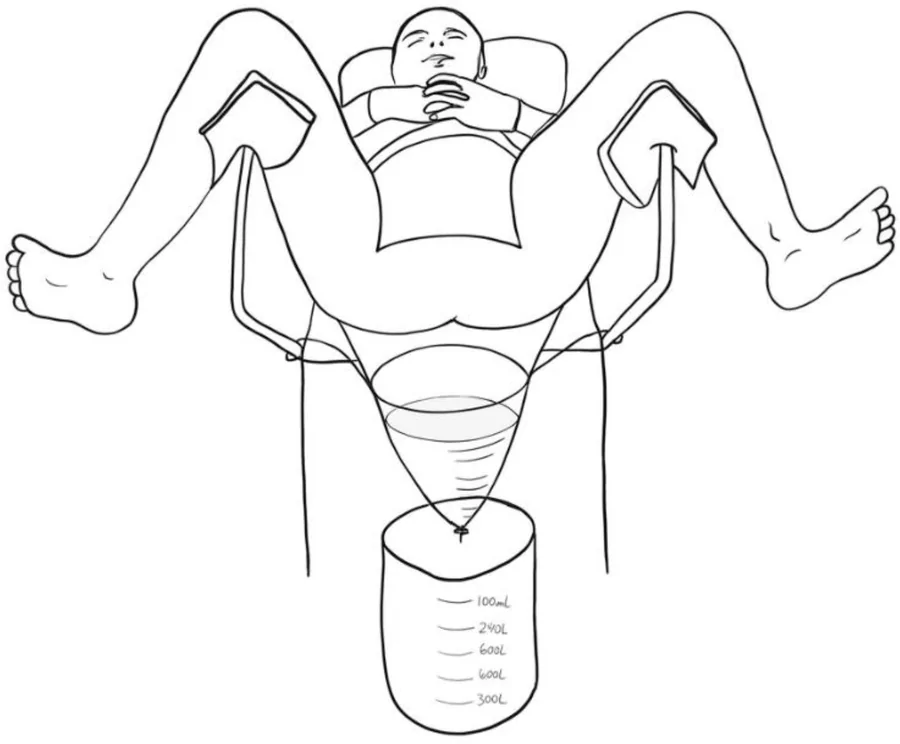
Schematic diagram of clinical use of precision distension fluid collection device.

The collection bag employs a dual fixation mechanism:

1. A traction rope connect to the lithotomy position footrest to ensure a secure connection.
2. Surface B has an adhesive portion, and the extended opening length of the bag allows the elongated part to be placed under the patient’s buttocks, providing dual fixation to prevent the bag from detaching due to the weight of the collected fluid.

### Original distension fluid collection device method

A 50 × 70 cm yellow plastic bag was used, with its tail end placed under the patient's buttocks. The handle of the plastic bag was tied with a slipknot, and its free end was draped into a garbage bin in the operating room to collect distension fluid. Postoperatively, the fluid output was calculated by measuring the liquid volume in the bin (Figure 3).

**Figure 3:**
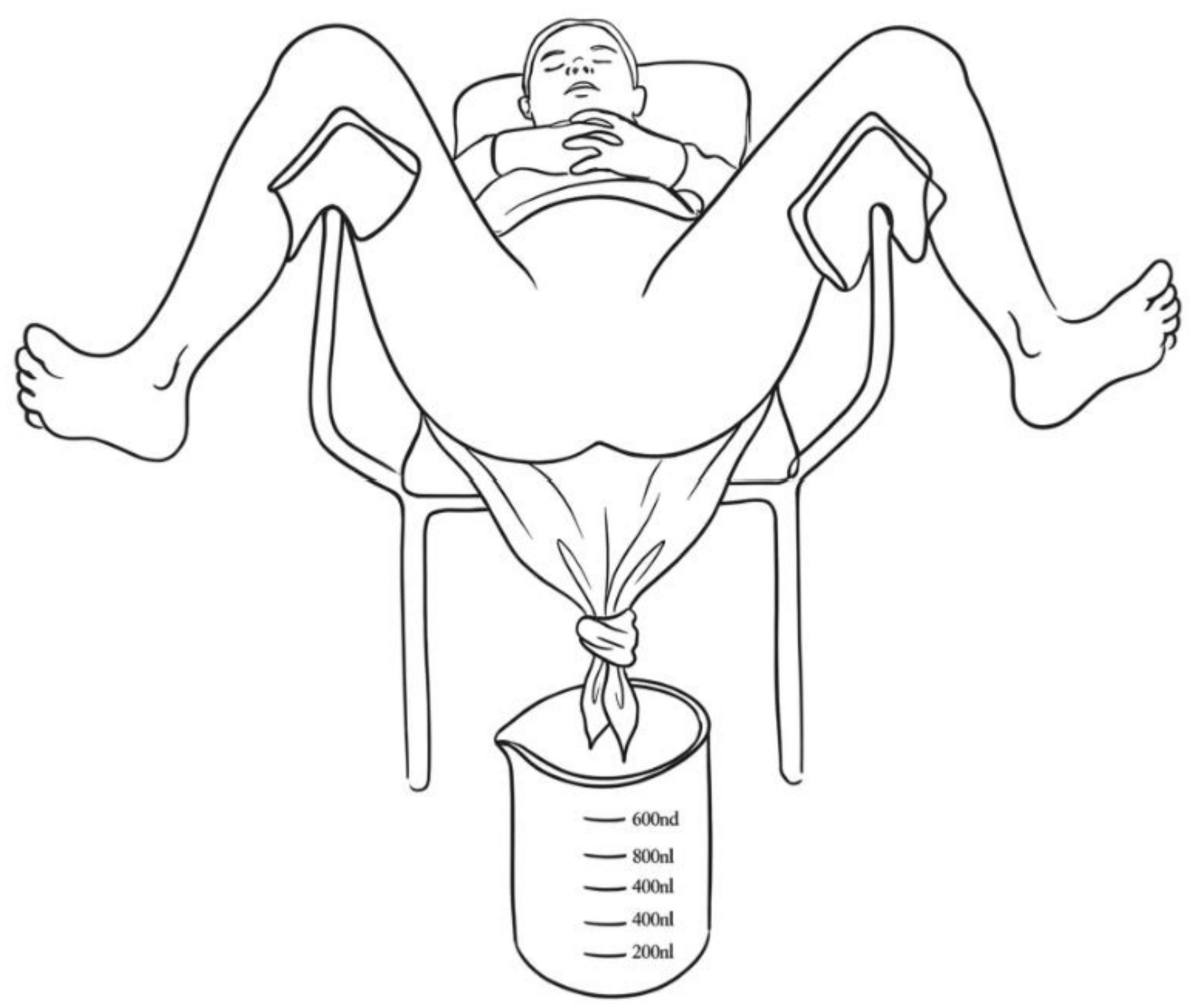
Schematic diagram of clinical use of original distension fluid collection device.

## Methods

### Ethics and registration

This study is a single-center, single-blind, randomized controlled trial. It was approved by the Clinical Research Ethics Committee of Lishui People's Hospital and registered in the Chinese Clinical Trial Registry (https://www.chictr.org.cn, No. ChiCTR2500101460, Apirl 25, 2025). The trial was conducted in accordance with the Declaration of Helsinki and Chinese clinical trial regulations. Informed consent was obtained from all patients prior to enrollment. Both patients and operating room cleaning staff were blinded to group assignments.

### Patient inclusion and exclusion criteria

Patients were eligible for inclusion if they were scheduled for elective hysteroscopic surgery, aged ≥18 years, classified as American Society of Anesthesiologists (ASA) physical status I or II, and had an anticipated operative time of less than 1 hour. Patients were excluded if they presented with severe organic dysfunction of the heart, brain, or kidneys; experienced an intraoperative change in the surgical approach; or had an anticipated preoperative requirement of ≤1,000 mL of distension fluid.

### Randomization and masking

Patients were enrolled preoperatively and randomly assigned in a 1:1 ratio to the Precision Collection Group (P Group) or the Control Group (C Group) using a computer-generated random number table in SPSS Statistics 25.0 (IBM Corp., Armonk, NY). The study was a randomized controlled single-blind trial, with patients blinded to their group assignment.

### Grouping and intervention

A computer-generated randomization sequence was used to allocate the precision group (Group P) and the control group (Group C) in a 1:1 ratio. To ensure strict allocation concealment, sequentially numbered, opaque, sealed envelopes were prepared according to the randomization sequence by an independent researcher who was not involved in patient recruitment or data collection. The envelopes were opened sequentially by the circulating nurse in the operating room only after the patient was admitted and the decision to perform hysteroscopic surgery was finalized. The precision group utilized a precision distension fluid collection device, while the control group used a standard distension fluid collection device. In this study, saline solution at 35–36 °C was used as the distension fluid for hysteroscopic surgery, with the distension pressure consistently set at 90 mmHg and a distension fluid flow rate of 300 mL/min. During the surgery, Ringer's solution was administered intravenously, with the infusion rate controlled at 2-4 mL/kg/h. After general anesthesia, the patient was placed in the lithotomy position, with a fluid collection device positioned under the buttocks to collect the distension fluid, and the lower end of the collection port was placed in a graduated measuring bucket. All procedures were performed by qualified gynecological surgeons under general anesthesia. Both groups collected and recorded the following data during hysteroscopic surgery: total distension fluid volume, collected fluid volume, operative time, complication rate, time required for operating room support staff to clean the surgical suite, and satisfaction scores of surgeons, nurses, and operating room support staff.

## Outcomes

### Primary outcome

The primary outcome is the distension fluid collection rate, defined as the volume of distension fluid collected divided by the total volume of fluid used during the procedure.

### Secondary outcomes

1. Incidence of adverse events:Includes the incidence of OHIAS, hypothermia, delirium, Uterine perforation, Infection and delayed emergence. 1)Hypothermia is defined as an ear temperature below 36 °C. 2)Delayed emergence is defined as the failure of a patient to regain consciousness 60 minutes after the discontinuation of anesthetic agents.
2. Electrolyte testing: Venous blood was collected at the end of the surgery for blood gas analysis. Changes in serum electrolytes and hemoglobin were determined by subtracting the preoperative measurements from the postoperative measurements. 1) Sodium Imbalance: The normal range for serum sodium concentration is 135–145 mmol/L. Hyponatremia is defined as a sodium level below 135 mmol/L, while hypernatremia is defined as a sodium level above 145 mmol/L. 2) Potassium Imbalance: The normal range for serum potassium concentration is 3.5–5.5 mmol/L. Hypokalemia is defined as a potassium level below 3.5 mmol/L, and hyperkalemia is defined as a potassium level above 5.5 mmol/L.
3. Operating room cleaning time: Recorded by the circulating nurse as the time required by operating room support staff to clean the operating room after hysteroscopic surgery.
4. Satisfaction of surgical personnel: Satisfaction surveys conducted among surgeons, nurses, and operating room support staff, scored on a scale of 1–5, where 5 = very satisfied, 4 = satisfied, 3 = neutral, 2 = dissatisfied, and 1 = very dissatisfied.

### Sample size and statistical analysis

A sample size calculation for a superiority trial was conducted using PASS 2011 software, informed by preliminary study findings. The distension fluid collection rate was estimated at 90% for the precision group and 50% for the control group, with a superiority margin of 0.20. The trial was designed with a one-sided Type I error rate of 0.025 and a statistical power of 0.90. Accounting for an anticipated 20% dropout rate, each group requires a cohort of 82 patients to ensure robust statistical power and accommodate potential loss to follow-up.

Statistical analysis was performed using SPSS Statistics 25.0 (IBM Corp., Armonk, NY). The Shapiro–Wilk test was applied to determine whether continuous variables were normally distributed. Normally distributed continuous variables were expressed as the mean ± standard deviation, and these data were analyzed using Student's t-test. Nonnormally distributed continuous variables were expressed as the medians and interquartile ranges (IQRs), and these data were analyzed using the Mann–Whitney U-test. Categorical outcomes are presented as frequency and percentage per study group and analyzed using the chi-square test or the Fisher's exact test, when appropriate. Ordinal categorical variables were analyzed using the Mann–Whitney U-test. Significance was set at *P* < 0.05.

## Results

The study included a total of 164 women, with 82 patients in each group (Figure 4). There were no differences in baseline characteristics between the two groups (Table 1). No significant differences were observed between the groups in terms of age, height, weight, BMI, ASA, inflow volume or level of serum sodium, potassium and hemoglobin(*P* > 0.05).

**Figure 4:**
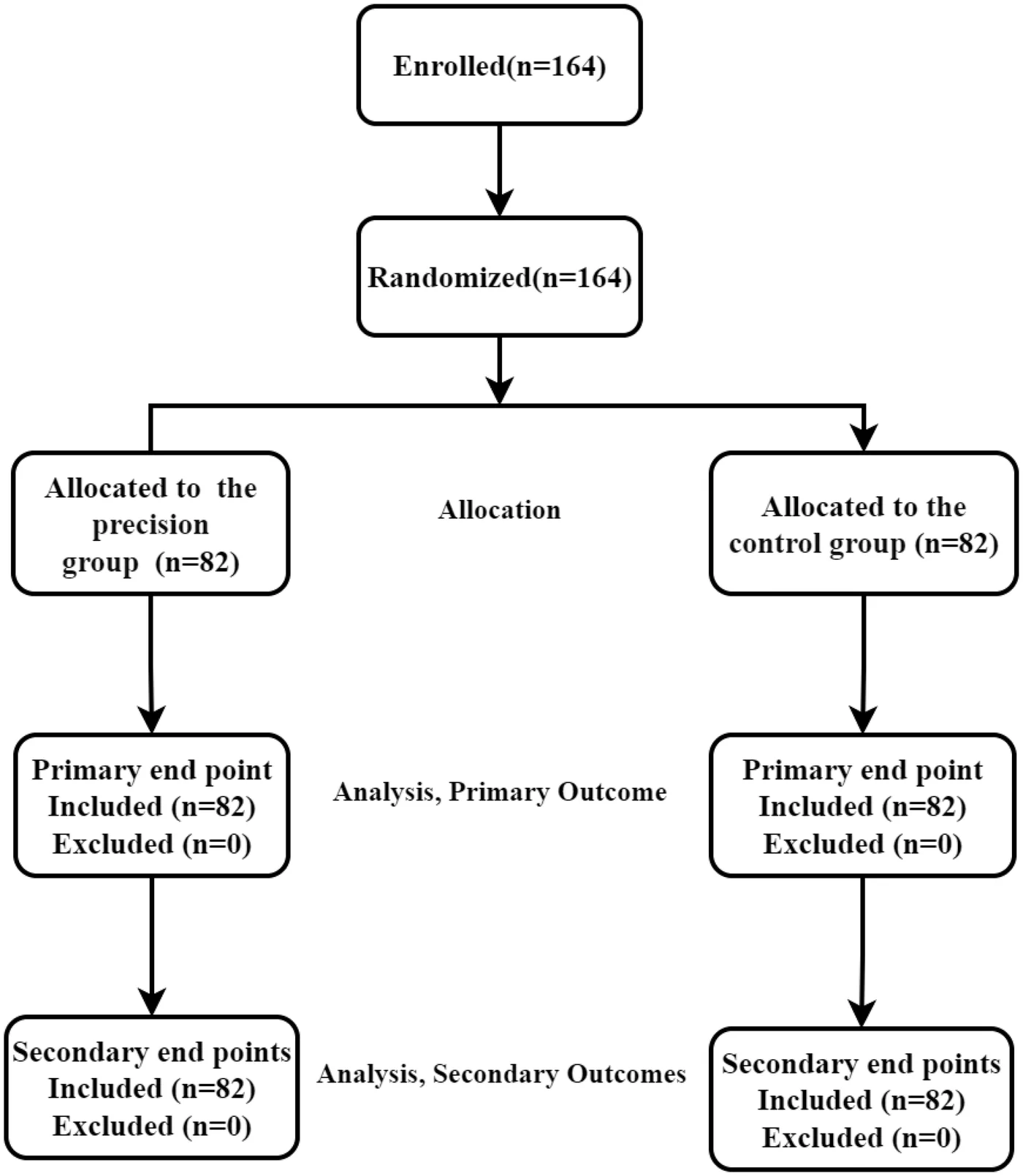
Flow diagram representing patient enrollment, group assignment, and analysis.

**Table 1:** Demographic characteristics and surgical data for each group.

Variables	Group P (*n* = 82)	Group C (*n* = 82)	*P*
Age, yr	38.4 ± 11.6	40.9 ± 10.9	0.099
Female, %	100	100	1.000
Height, cm	159.2 ± 4.2	158.6 ± 4.1	0.766
Weight, kg	58.8 ± 7.8	57.8 ± 7.0	0.392
Body mass index, kg/m2	23.2 ± 2.9	22.9 ± 2.6	0.599
Serum sodium (mmol/L)	141.2 ± 2.5	140.6 ± 2.7	0.108
Serum potassium (mmol/L)	3.75 ± 0.24	3.82 ± 0.38	0.325
Hemoglobin (g/dL)	11.1 ± 1.2	10.9 ± 1.3	0.146
ASA			0.429
I	45 (54.9)	50 (61.0)	
II	37 (45.1)	32 (39.0)	
Duration of operation (min)	30.2 ± 1.3	28.5 ± 1.5	0.217
Inflow volume (mL)	4,804.6 ± 1,272.0	4,781.0 ± 1,644.3	0.918
Collected volume(mL)	4,548.6 ± 1,216.7	2,314.3 ± 861.2	<0.001
Primary surgical procedure, n(%):			0.421
Lesion Resection	36 (43.9)	30 (36.6)	
Diagnostic Hysteroscopy	33 (40.2)	38 (46.3)	
Hysteroscopic Myomectomy	8 (9.8)	7 (8.5)	
Removal of Retained Products of Conception (RPOC)	5 (6.1)	7(8.5)	

Data indicate the mean ± SD or n (%). Group P, the group of precision distension fluid collection device; Group C, the group of original distension fluid collection device; ASA, American Society of Anesthesiologists.; Lesion Resection, in this study primarily refers to superficial endometrial lesions and polypectomies involving minimal myometrial disruption.

There were no significant statistical differences in surgical duration or procedure types between the two groups. Compared to the control group, the precise fluid collection group exhibited a significantly higher fluid collection rate (48% vs. 95%, *P* < 0.001). Additionally, the postoperative cleanup time for the precise fluid collection group was significantly reduced (6.4 min vs. 4.1 min, *P* < 0.001). Meanwhile, the satisfaction of the operating room team was markedly improved in the precise fluid collection group (*P* < 0.001). No patients in either group experienced severe perioperative complications related to the surgery. In the control group, 7 patients developed postoperative hypothermia, while 3 patients in the experimental group had hypothermia, and 1 patient in the experimental group experienced uterine perforation. The length of stay in the Post-Anesthesia Care Unit (PACU) was comparable between the two groups (*P* > 0.05). Comparison of preoperative and postoperative electrolyte levels showed a slight decrease in both groups compared to preoperative levels, but the reduction was mild, with no statistical difference between the groups (*P* > 0.05). No severe electrolyte imbalances were observed in either group (Table 2).

**Table 2:** Collection-related outcomes.

Outcomes	Group P (*n* = 82)	Group C (*n* = 82)	*P*
Distension fluid recovery rate ^a^	0.95 ± 0.03	0.48 ± 0.07	＜0.001
Cleaning Time(min) ^a^	4.1 ± 1.1	6.4 ± 1.5	＜0.001
PACU stay	29.7 ± 6.0	30.7 ± 6.4	0.365
Satisfaction of Surgical Personnel^a^			
Surgeon			＜0.001
1	0 (0)	0 (0)	
2	0 (0)	0 (0)	
3	0 (0)	0 (0)	
4	10 (12.2)	51 (62.2)	
5	72 (87.8)	31 (37.8)	
Nurse			＜0.001
1	0 (0)	0 (0)	
2	0 (0)	0 (0)	
3	0 (0)	6 (7.3)	
4	15 (18.3)	70 (85.4)	
5	67 (81.7)	6 (7.3)	
Support Staff			＜0.001
1	0 (0)	0 (0)	
2	0 (0)	0 (0)	
3	2 (2.4)	39 (47.6)	
4	20 (24.4)	38 (46.3)	
5	60 (73.2)	5 (6.1)	
Incidence of Adverse Events (n,%)			
OHIAS	0 (0)	0 (0)	1.000
Hypothermia	3 (3.7)	7 (8.5)	0.192
Delirium	0 (0)	0 (0)	1.000
Delayed emergence	0 (0)	0 (0)	1.000
Uterine perforation	1 (1.2)	0 (0)	1.000
Infection	0 (0)	0 (0)	1.000
Electrolyte imbalance:			
Decline in serum Na + (mmol/L)	1.8 ± 0.83	2.0 ± 0.85	0.078
Decline in serum K + (mmol/L)	0.18 ± 0.08	0.20 ± 0.10	0.088
Decline in postop Hb (g/dL)	0.40 ± 0.19	0.45 ± 0.23	0.243

a Indicates *P* < 0.05 between the groups. PACU, post-anesthesia care unit; OHIAS, operative hysteroscopy intravascular absorption syndrome.

## Discussion

The results of this study showed that the precision distension fluid collection device successfully increased the recovery rate of distension fluid during hysteroscopic surgery from 48% in the control group to a remarkable 95% in the experimental group. By enabling precise fluid collection and effectively preventing fluid spillage, this efficient collection device not only shortened postoperative operating room cleanup time but also improved the satisfaction of the surgical team, demonstrating promising potential for broad clinical application.

Our research rationale and core conclusions align with a prior study by Marshburn, who developed a total capture drape designed to minimize unabsorbed fluid leakage onto the operating room floor or blankets, thereby enhancing patient safety and surgical care quality (23). Our device introduces targeted design modifications to address specific practical limitations observed with their drape. First, the integration of a calibrated measurement scale at the bottom of the bag allows for direct and real-time quantification of the collected volume. Second, to address the practical challenge of device displacement caused by frequent hysteroscopic manipulations, our device incorporates symmetrical securement ropes on both sides of the collection bag. This structural modification successfully anchors the apparatus, thereby enhancing both physical stability and operational reliability throughout the procedure.

In the present study, the overall incidence of perioperative complications was consistent with the 0.2%–2.7% range reported in previous literature (2, 3, 6, 8). The incidence of postoperative hypothermia was 8.5% in the control group and 3.7% in the experimental group. In hysteroscopic surgery, hypothermia is mainly related to operating room temperature, patient-specific differences, and fluid exposure. In conventional practice, distension fluid may spill and wet the drapes, increasing heat loss. In contrast, this device can improve the collection rate of distension fluid, reduce drape wetting, and shorten the duration of patient contact with a cold, wet environment, thereby helping to reduce the incidence of hypothermia. Additionally, the single case of uterine perforation in the experimental group occurred during a hysteroscopic myomectomy, a complex operative procedure associated with an inherently higher baseline risk of perforation (2, 24, 25). No intraoperative OHIAS events were observed in this study, which may be attributed to two factors. First, the relatively limited sample size of our study reduced the statistical power to detect rare complications such as severe fluid overload. Second, although there is currently no high-quality evidence directly linking specific procedure types to the risk of OHIAS, the extent of intravascular fluid absorption is inherently driven primarily by operative duration and the depth of myometrial resection (26). In this study, the vast majority of included cases were low-risk procedures, such as diagnostic hysteroscopy and superficial polypectomy, which typically involve minimal myometrial injury and short operative times, thereby fundamentally reducing the baseline risk of excessive fluid absorption. Therefore, for high-risk complex hysteroscopic surgeries, precise tracking of fluid deficit is of even greater clinical importance. Alongside volume-safety parameters, postoperative serum sodium and potassium levels showed a slight decrease relative to baseline in both groups, yet remained clinically benign without requiring medical intervention. This minor fluctuation potentially stems from preoperative fluid restriction alongside modest fluid intravasation. Crucially, while using isotonic saline avoids profound metabolic derangements, these subtle electrolyte shifts emphasize the value of continuous perioperative electrolyte surveillance (27, 28).

Given the clinical severity of fluid overload, precise monitoring of the fluid deficit remains the cornerstone of prevention. To this end, current guidelines from the AAGL recommend terminating hysteroscopic procedures immediately if the fluid deficit reaches specific safety thresholds, such as 2,500 mL for isotonic media (10, 13). Although automated, electronic pump-based systems with real-time tracking are the preferred approach, their high costs often limit implementation in resource-limited or basic-level clinical settings where the rudimentary plastic bag method remains the practical standard. Our device addresses this practical gap, offering an affordable and accessible volumetric monitoring alternative that assists clinicians in adhering to safety guidelines when automated infrastructure is unavailable.

This study is subject to several limitations. First, due to the nature of the interventions, blinding the surgical team was impossible, which may have introduced performance and detection biases regarding subjective secondary outcomes like staff satisfaction scores. Second, our institution currently lacks automated fluid management systems; thus, a traditional plastic bag method remains the practical standard of care and was used as the comparator, which may limit the generalizability of our findings to advanced medical centers. Third, patients with an anticipated distension volume of ≤ 1,000 mL were excluded, limiting the generalizability of our findings to low-volume outpatient diagnostic hysteroscopies. Fourth, the relatively small sample size and limited proportion of high-risk operative cases necessitate larger-scale trials to validate the device's specific efficacy in preventing fluid overload during high-risk procedures. Finally, the collected distension fluid was mixed with urine and blood, which may have introduced minor variations into the recovery rate data. Future large-scale studies incorporating complex or prolonged procedures are warranted to further evaluate the clinical utility of this device.

## Conclusion

The precision distension fluid collection device effectively enhances the collection rate of hysteroscopic distension fluid, reduces risks associated with improper fluid management, shortens operating room turnover time, and improves the satisfaction of the surgical team.

## Data Availability

The original contributions presented in the study are included in the article/Supplementary Material, further inquiries can be directed to the corresponding authors.
